# Changes of NLRP3 in serum and cerebrospinal fluid of patients after moderate to severe traumatic brain injury and their predictive values for prognosis

**DOI:** 10.1111/cns.70009

**Published:** 2024-09-20

**Authors:** Wei Chen, Zhigang Wang, Gengfan Ye, Guangyao Zhu, Shiwei Li, Pandi Chen, Hongcai Wang, Shufeng Zou, Maosong Chen

**Affiliations:** ^1^ Department of Neurosurgery The Affiliated Lihuili Hospital of Ningbo University Ningbo Zhejiang China; ^2^ Department of Neurosurgery, Shanghai East Hospital, School of Medicine Tongji University Shanghai China; ^3^ Department of Neurosurgery The First Affiliated Hospital of Nanchang University Nanchang Jiangxi China

**Keywords:** biomarker, CSF, NLRP3, outcome, TBI

## Abstract

**Background:**

Traumatic brain injury (TBI) remains a major concern for global health. Recent studies have suggested the role of NOD‐like receptor pyrin domain‐containing protein 3 (NLRP3), an inflammatory marker, in the cerebrospinal fluid (CSF) and serum as potential indicators of TBI prognosis. The objective of the study was to characterize NLRP3 as a clinically applicable tool for predicting the outcomes of TBI patients.

**Methods:**

A total of 270 patients with moderate to severe TBI were included in this retrospective analysis. Serum and CSF samples were collected at 1‐, 3‐, 7‐, and 21‐day post‐injury to measure NLRP3 levels. The prognosis of patients was evaluated after 3 months using the Glasgow Outcome Scale (GOS). Patients were categorized into good prognosis (GOS score >3) and poor prognosis (GOS score ≤3) groups. The relationship between NLRP3 levels and prognosis was analyzed.

**Results:**

Patients with poor prognosis had significantly elevated NLRP3 levels in their serum on days 1 and 3 post‐injury compared with those with a good prognosis. The difference was more pronounced during these early days compared with days 7 and 21. However, NLRP3 levels in CSF consistently showed a large difference between the two groups throughout the observation period. Receiver operating characteristic analysis revealed that the level of NLRP3 in the CSF on day 3 post‐injury had the highest predictive value for prognosis, with an area under the curve of 0.83, followed by the level of NLRP3 in the serum on day 3 post‐injury.

**Conclusions:**

The levels of NLRP3, especially in the CSF on day 3 post‐injury, can serve as a potential biomarker for predicting prognosis in moderate to severe TBI patients. Early measurement of NLRP3 levels can provide valuable insights into patient outcomes and guide therapeutic strategies.

## Graphical abstract

The study has provided that Levels of NLRP3, especially in the CSF on day 3 post‐injury, could serve as a potential biomarker for predicting prognosis in moderate to severe TBI patients. Early measurement of NLRP3 levels could provide valuable insights into patient outcomes and guide therapeutic strategies.

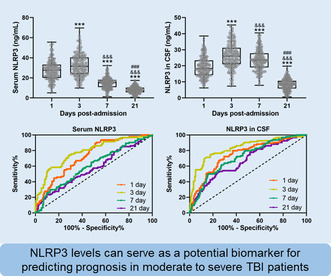

## 1 INTRODUCTION

Traumatic brain injury (TBI) is a damage to brain tissues caused by trauma. It significantly affects human health and is one of the leading causes of death worldwide. It is shown that the annual death rate from TBI in China is 12.99 per 100,000 population.^1^ A large data analysis from 16 European countries showed that the number of hospitalizations due to TBI was 287.2 per 100,000 population,^2^ with a mortality rate of 11.7%. Although the death rate of TBI has decreased in recent years due to the establishment of specialized neurological intensive care units (ICUs)^3^ and the implementation of evidence‐based TBI guidelines,^4^ a significant number of survivors still suffer from various neurological impairments caused by neuronal damage, including thinking, language, learning, emotional and cognitive‐behavioral disorders, and even psychiatric disorders.^5^ Hence, identifying biomarkers predictive of TBI severity and outcome is essential for improving the outcomes of TBI and facilitate treatment planning.^6^

The inflammasome is a multi‐protein complex present in the cytoplasm. The NOD‐like receptor pyrin domain‐containing protein 3 (NLRP3) is a major component of the inflammasome,^7^ which is composed of NLRP3, apoptosis‐associated speck‐like protein (ASC), and caspase 1, and plays a role in the body's innate immune response. Normally, NLRP3 activity in the body is low, but it can activate the apoptotic pathway and mediate inflammatory reactions when stimulated, even triggering an inflammatory cascade.^8^

Despite previous studies showing an upregulation of inflammatory factors following TBI and their correlations to clinical outcomes,^9^, ^10^, ^11^ a single inflammatory factor has yet to be established as a clinically applicable tool for patient prognosis and therapeutic target development. In contrast, NLRP3 can mediate the expression of various inflammatory factors, our current focus is on the expression of NLRP3, with the goal of developing NLRP3 as a feasible tool for prognosis among TBI patients.

Emerging studies have reported the use of NLRP3 as a biomarker for neurological diseases.^12^ For example, elevated levels of NLRP3 have been observed in patients with Alzheimer's disease (AD),^13^ multiple sclerosis (MS),^14^ and other neurodegenerative conditions, suggesting its potential involvement in the pathogenesis of these disorders. Additionally, its detectability in both serum and CSF makes it an amenable biomarker for diagnostic procedures.^15^ In studying the diagnostic utility of NLRP3 in AD, studies have shown that the NLRP3 inflammasome activation is prevalent in the brains of AD patients, which is associated with the production of IL‐1β, a pro‐inflammatory cytokine.^13^ In MS, decreased NLRP3 inflammasome activation has been reported in MS lesions. Notably, NLRP3 levels in the CSF of MS patients correlate with disease severity, making it a potential tool for prognosis and monitoring.^14^ Post‐mortem studies have revealed that NLRP3 inflammasome components are upregulated in the substantia nigra of patients with Parkinson's disease (PD).^16^ Moreover, animal models of PD have demonstrated that blocking NLRP3 can attenuate symptoms, emphasizing its potential therapeutic significance.^17^ Despite recent studies on the use of NLRP3 as a diagnostic biomarker for TBI,^18^ the correlation of NLRP3 to TBI severity assessment and prognosis remains to be demonstrated in clinical settings.

The aim is to study the changes in NLRP3 in CSF and serum after TBI and analyze its value for the prognosis of TBI patients, with the goal of characterizing NLRP3 as a feasible clinical tool for managing TBI patients. Our study included a total of 270 and analyzed levels of NLRP3 in CSF and serum in association with disease severity and prognosis. Receiver operating characteristic (ROC) analysis was used for assessing predictive value of the biomarker. Our study could provide evidence on the performance of NLRP3 as a predictive biomarker for improving manage of TBI patients.

## 2 METHODS

### 2.1 Patients

The study was approved by the ethics committee of the Affiliated Lihuili Hospital of Ningbo University, and written consent was derived from the participants. The study was performed in strict accordance with the Declaration of Helsinki, Ethical Principles for Medical Research Involving Human Subjects.

Inclusion Criteria:

Diagnosis of traumatic brain injury TBI according to the diagnostic criteria for brain injury established by the 4th Cerebrovascular Disease Academic Conference in 1995; confirmed by head CT or MRI; age ≥ 18 years; admitted for treatment within 12 h after injury; moderate and severe TBI patients; informed consent from the patient and their families.

Exclusion Criteria:

Previous history of head trauma or other intracranial space‐occupying diseases, cerebral infarction, etc.; serious heart, lung, liver, kidney, or other vital organ diseases; concurrent malignant tumors. Excluding those who die within 21 days of admission.

### 2.2 TBI severity assessment

The severity of TBI was categorized into moderate (9–12) and severe (3–8) based on the Glasgow Coma Scale (GCS) score.^19^ Briefly, after admitting patients for treatment, follow‐up for 3 months and evaluate all patient prognoses using the Glasgow Outcome Scale (GOS). At the end of the follow‐up, a GOS score >3 is considered a good prognosis (good prognosis group), and ≤3 is a poor prognosis (poor prognosis group). Five is good recovery and normal life with minor defects; 4 is mild disability but can live independently and work under protection; 3 is severe disability, awake but requires daily care; 2 is a vegetative state with minimal responses (e.g., following sleep/wake cycles, can open eyes); 1 is death.

Obtain patient cerebrospinal fluid and fasting serum on days 1, 3, 7, and 21 after admission, and measure the levels of NLRP3 in cerebrospinal fluid and serum. Use Enzyme‐linked immunosorbent assay (ELISA) to determine the concentration of NLRP3 in cerebrospinal fluid and serum. The kit is purchased from Wuhan Fine Bio, product number EH4202, detection range: 0.781–50 ng/mL, and sensitivity: 0.469 ng/mL.

### 2.3 Statistical analysis

The sample size was determined by established statistical power analysis. Estimates of effect size and standard deviation were based on the existing literature. The data were shown with mean ± SD or *n*%. The comparison between categorical variables was done by Chi‐square or Fisher's exact test. Kolmogorov–Smirnov test was used to test normality of the continuous variables. Unpaired *t* test with Welch's correction and Brown‐Forsythe ANOVA test followed by a Games‐Howell's multiple comparisons test were used to calculate the *p* values.

## 3 RESULTS

### 3.1 Study design and baseline characteristics

In this study, we obtained serum and CSF samples from a total of 270 patients with moderate to severe TBI on days 1, 3, 7, and 21 after their TBI admission. After patients were admitted for treatment, they were followed up for 3 months. The GOS was used to evaluate the prognosis of all patients. The baseline characteristics of the patients are shown in Table 1. At the end of the follow‐up period, a GOS score of >3 was considered a good prognosis (Good prognosis group), whereas a score of ≤3 was deemed a poor prognosis (Poor prognosis group). Among them, there were 176 cases in the good prognosis group and 94 cases in the poor prognosis group. Analyzing the baseline data between the two groups, we found significant differences in aspects such as age, severity of TBI, occurrence of cerebral contusions and lacerations, intracranial hematoma, and epidural hematoma.

**TABLE 1:** Baseline clinical characteristics of moderate to severe traumatic brain injury (TBI) patients with good or poor prognosis.

Characteristics	Good (*n* = 176)	Poor (*n* = 94)	*p* value
Age (years)			
<60	119 (67.6%)	48 (51.1%)	0.009
≥60	57 (32.4%)	46 (48.9%)	
Gender (*n*, %)			
Male	103 (58.5%)	50 (53.2%)	0.440
Female	73 (41.5%)	44 (46.8%)	
Severity at admission			
Moderate	115 (65.3%)	42 (44.7%)	0.001
Severe	61 (34.7%)	52 (55.3%)	
Cause of injury			
Accident	107 (60.8%)	49 (52.1%)	0.211
Falling	49 (27.8%)	36 (38.3%)	
Others	20 (11.4%)	9 (9.6%)	
Diabetes mellitus			
Yes	20 (11.4%)	15 (15.9%)	0.342
No	156 (88.6%)	79 (84.1%)	
Hypertension			
Yes	31 (17.6%)	24 (25.5%)	0.153
No	145 (82.4%)	70 (74.5%)	
Cerebral contusion and laceration			
Yes	53 (30.1%)	41 (43.6%)	0.032
No	123 (69.9%)	53 (56.4%)	
Intracerebral hematoma			
Yes	29 (16.5%)	29 (30.9%)	0.008
No	147 (83.5%)	65 (69.1%)	
Epidural hematoma			
Yes	52 (29.5%)	45 (47.9%)	0.003
No	124 (70.5%)	49 (52.1%)	

The data are presented as *n*%. The comparisons of data between the two groups were done by Fisher's exact test or Chi‐square test.

### 3.2 Temporal changes of NLRP3 in serum and cerebrospinal fluid of TBI patients

**FIGURE 1:**
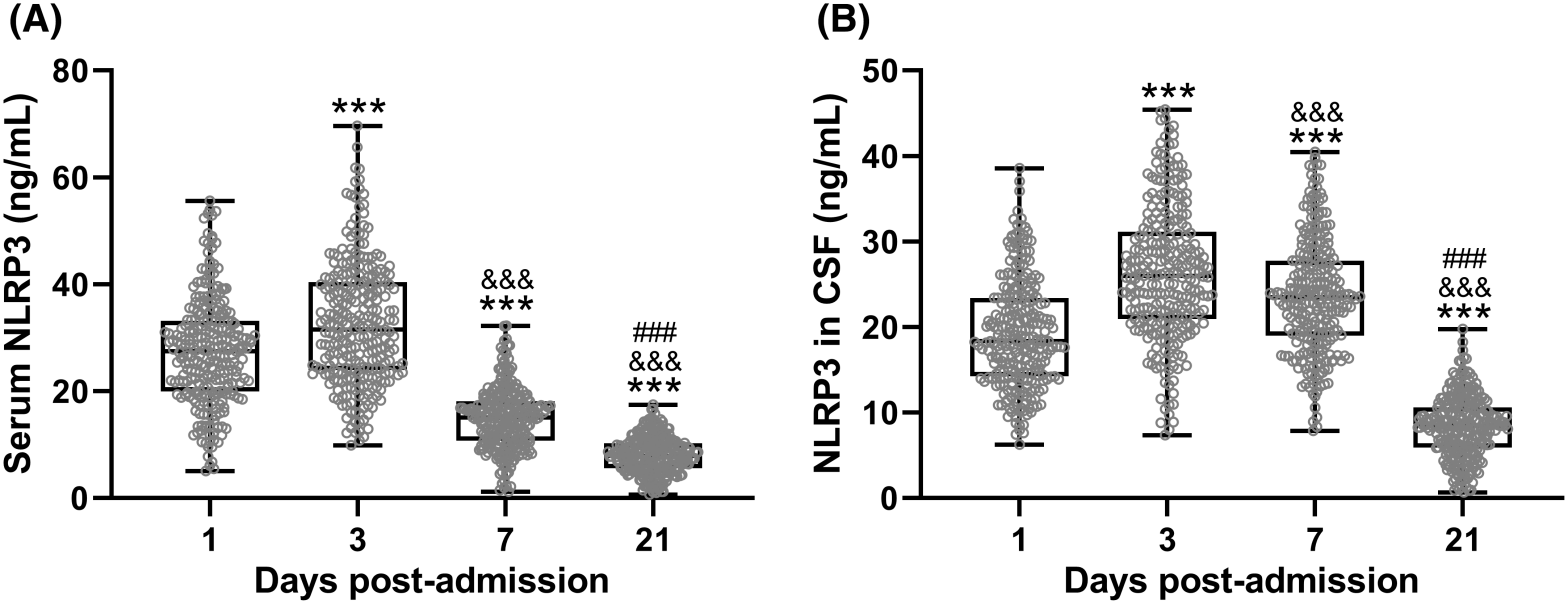
Changes of NLRP3 in serum (A) and cerebrospinal fluid (CSF, B) of patients after moderate to severe traumatic brain injury. *n* = 270. The data were shown with mean ± SD. ***p < 0.001 compared with 1‐day post‐admission. ^&&&^ *p* < 0.001 compared with 3 days post‐admission. ^###^ *p* < 0.001 compared with 7 days post‐admission. Brown‐Forsythe ANOVA test followed by a Games‐Howell's multiple comparisons test.

We analyzed the changes in the concentration of NLRP3 in the serum and cerebrospinal fluid (CSF) of patients with moderate to severe TBI at different times. Figure 1 demonstrates the temporal changes of NLRP3 in serum (Figure 1A 1 day: 27.31 ± 9.92 ng/mL, 3 days: 32.55 ± 11.35 ng/mL, 7 days: 14.92 ± 5.75 ng/mL, 21 days: 8.01 ± 3.37 ng/mL) and CSF (Figure 1B 1 day: 19.21 ± 6.28 ng/mL, 3 days: 26.32 ± 7.76 ng/mL, 7 days: 23.64 ± 6.64 ng/mL, 21 days: 8.35 ± 3.58 ng/mL). It can be observed that the concentration of NLRP3 in both serum and CSF reached its peak 3 days after the injury in patients with moderate to severe TBI. It then began to decline, with the serum concentration of NLRP3 already dropping to a low level by the 7th day, lower than the level on the first day post‐injury. However, the level of NLRP3 in the cerebrospinal fluid remained higher on the 7th day post‐injury compared with the first day. By the 21st day post‐injury, the levels of NLRP3 in both cerebrospinal fluid and serum were relatively low.

### 3.3 Severe TBI is characterized increased levels of NLRP3 in serum and CSF

**FIGURE 2:**
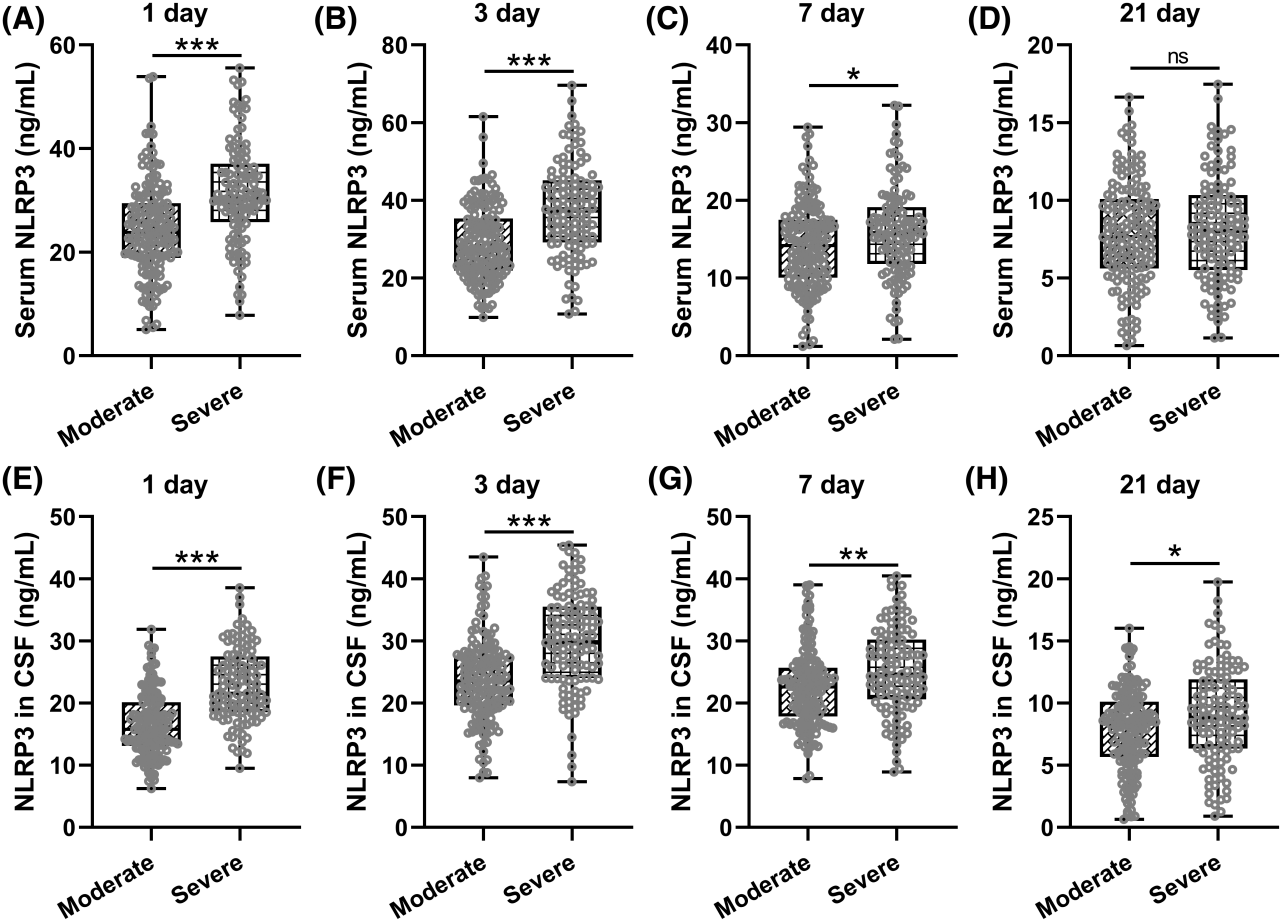
Comparisons of serum NLRP3 at 1 (A), 3 (B), 7 (C) and 21 (D) days post‐admission between patients with moderate and severe traumatic brain injury. Comparisons of NLRP3 in cerebrospinal fluid (CSF) at 1 (E), 3 (F), 7 (G) and 21 (H) days post‐admission between patients with moderate and severe traumatic brain injury. *n* = 157 for moderate and *n* = 113 for severe. *p < 0.05, **p < 0.01, ***p < 0.001.

Figure 2 analyzes the relationship between the levels of NLRP3 in the serum and CSF of patients with moderate to severe TBI on days 1, 3, 7, and 21 after their TBI admission and the severity of the disease. It is evident that the difference in serum NLRP3 levels between moderate and severe patients was most pronounced on days 1 and 3 post‐injury, with severe patients having a higher concentration of NLRP3 in their serum (Figure 2A–D 1 day: 24.34 ± 8.92 ng/mL for moderate and 31.44 ± 9.79 ng/mL for severe; 3 days: 28.90 ± 9.45 ng/mL for moderate and 37.61 ± 11.86 ng/mL for severe; 7 days: 14.16 ± 5.48 ng/mL for moderate and 15.98 ± 5.98 ng/mL for severe; 21 days: 7.87 ± 3.33 ng/mL for moderate and 8.19 ± 3.43 ng/mL for severe). By the 7th day post‐injury, the difference between the two groups began to decrease, and by the 21st day, there was no significant difference in serum NLRP3 levels between moderate and severe patients.

The levels of NLRP3 in the CSF demonstrated distinctive patterns (Figure 2E–H 1 day: 16.58 ± 5.11 ng/mL for moderate and 22.86 ± 5.94 ng/mL for severe; 3 days: 23.69 ± 6.61 ng/mL for moderate and 29.98 ± 7.76 ng/mL for severe; 7 days: 22.52 ± 6.21 ng/mL for moderate and 25.20 ± 6.92 ng/mL for severe; 21 days: 7.86 ± 3.25 ng/mL for moderate and 9.02 ± 3.91 ng/mL for severe) than that in serum. The difference in NLRP3 levels in CSF between moderate and severe patients was most significant on days 1 and 3 post‐injury, with the levels being higher in severe patients. By the 7th day post‐injury, the difference between the two groups began to decrease, but by the 21st day, there was still a significant difference between the two groups.

### 3.4 Increased levels of NLRP3 in serum and CSF are associated with poor outcomes for TBI patients

**FIGURE 3:**
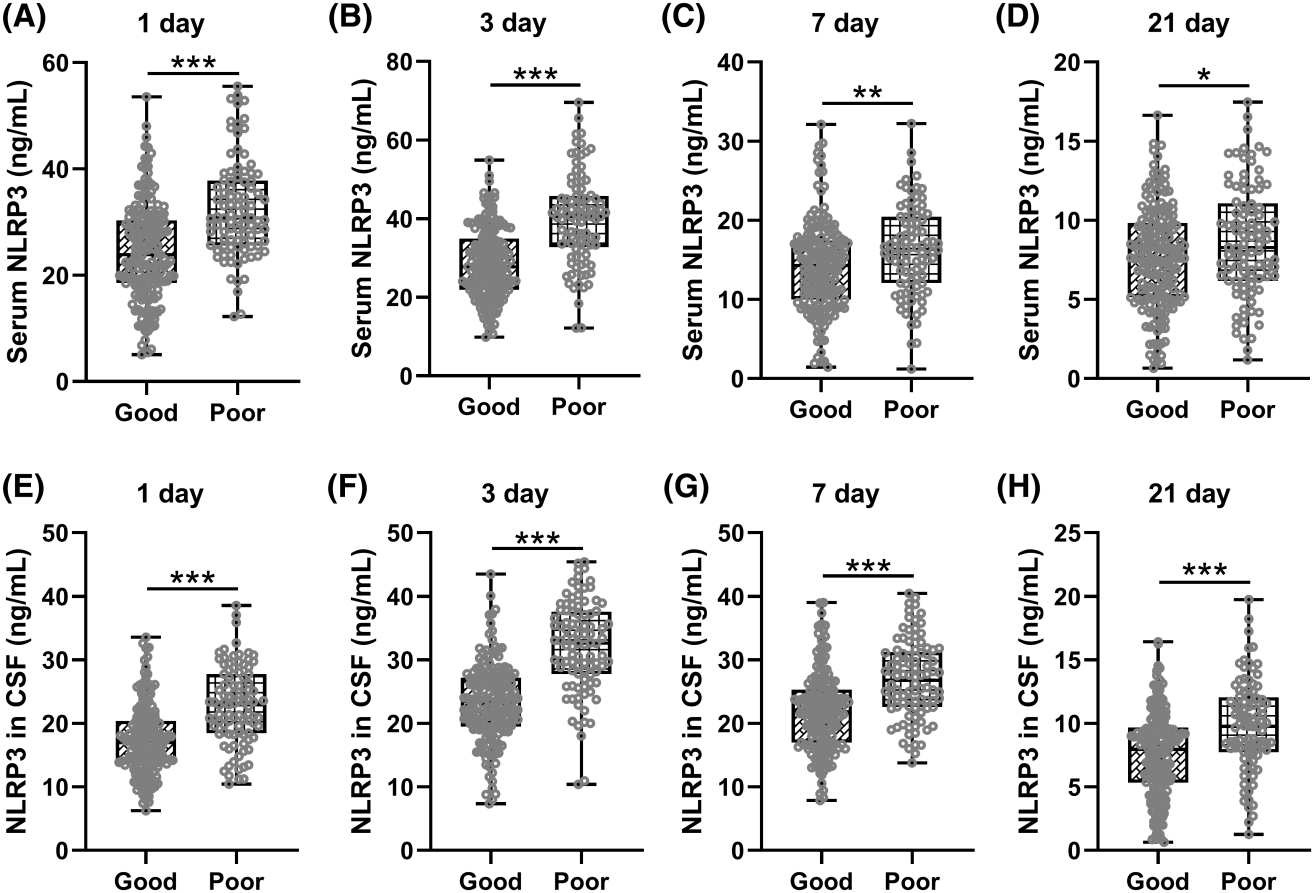
Comparisons of serum NLRP3 at 1 (A), 3 (B), 7 (C) and 21 (D) days post‐admission in moderate to severe traumatic brain injury patients with good or poor prognosis. Comparisons of NLRP3 in cerebrospinal fluid (CSF) at 1 (E), 3 (F), 7 (G) and 21 (H) days post‐admission in moderate to severe traumatic brain injury patients with good or poor prognosis. *n* = 176 for good prognosis and *n* = 94 for poor prognosis. *p < 0.05, **p < 0.01, ***p < 0.001.

As shown in Figure 3, both serum (Figure 3A–D 1 day: 24.54 ± 9.15 ng/mL for good and 32.50 ± 9.22 ng/mL for poor; 3 days: 28.95 ± 9.10 ng/mL for good and 39.95 ± 11.47 ng/mL for poor; 7 days: 14.23 ± 5.70 ng/mL for good and 16.21 ± 5.65 ng/mL for poor; 21 days: 7.67 ± 3.20 ng/mL for good and 8.65 ± 3.59 ng/mL for poor) and CSF (Figure 3E–H 1 day: 17.31 ± 5.48 ng/mL for good and 22.76 ± 6.15 ng/mL for poor; 3 days: 23.23 ± 6.12 ng/mL for good and 32.11 ± 7.18 ng/mL for poor; 7 days: 21.91 ± 6.24 ng/mL for good and 26.89 ± 6.16 ng/mL for poor; 21 days: 7.58 ± 3.34 ng/mL for good and 9.78 ± 3.59 ng/mL for poor) NLRP3 levels were higher in patients with a poor clinical outcome at 3 months follow‐up. Patients with a poorer prognosis had a more significant difference in the serum NLRP3 levels at 1‐ and 3‐day post‐injury compared with patients with a good prognosis than at 7‐ and 21‐day post‐injury. However, the difference in the CSF levels of NLRP3 between the good and poor prognosis groups was consistently pronounced both in serum and in CSF.

### 3.5 Values of serum and CSF NLRP3 in predicting TBI severity and outcome

**FIGURE 4:**
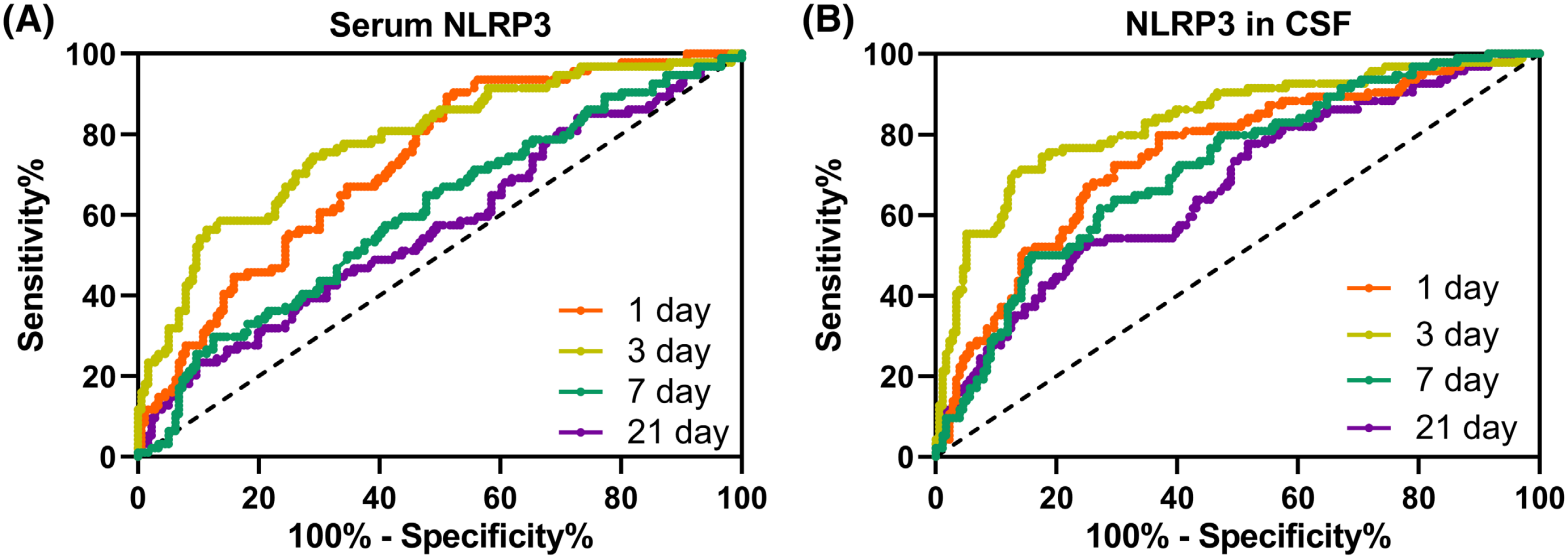
ROC analysis of predicative values of NLRP3 in serum (A) and cerebrospinal fluid (CSF, B) at 1‐, 3‐, 7‐, and 21‐days post‐admission for prognosis in patients with moderate and severe traumatic brain injury.

The predictive value of NLRP3 in TBI was shown in ROC analysis (Figure 4). Both serum (Figure 4A) and CSF (Figure 4B) NLRP3 demonstrated a high area under the curve (AUC), with the levels at 1 and 3 days showing the most prominent predictive capability. Table 2 shows the cut‐off value, AUC, sensitivity, and specificity based on the Youden's Index. It can be seen that for patients with moderate to severe TBI, the level of NLRP3 in the CSF at 3 days post‐injury holds the most predictive value for prognosis, with an AUC of 0.83 (0.78–0.89), the sensitivity is 70.21% and the specificity is 86.93%. This is followed by the level of NLRP3 in the serum 3 days post‐injury.

**TABLE 2:** Predictive values in ROC analysis.

	Cut‐off value	AUC (95% CI)	*p*	Sensitivity (%)	Specificity (%)	Youden index
Serum NLRP3‐1 day post‐admission	23.23 ng/mL	0.73 (0.67–0.79)	<0.001	89.36	48.86	0.38
Serum NLRP3‐3 days post‐admission	33.26 ng/mL	0.78 (0.73–0.84)	<0.001	74.47	71.02	0.45
Serum NLRP3‐7 days post‐admission	19.51 ng/mL	0.61 (0.53–0.68)	0.004	29.79	87.50	0.17
Serum NLRP3‐21 days post‐admission	11.90 ng/mL	0.57 (0.49–0.64)	0.058	23.40	89.77	0.13
NLRP3 in CSF‐1 day post‐admission	18.18 ng/mL	0.75 (0.69–0.81)	<0.001	79.79	63.07	0.43
NLRP3 in CSF‐3 days post‐admission	28.96 ng/mL	0.83 (0.78 to 0.89)	<0.001	70.21	86.93	0.57
NLRP3 in CSF‐7 days post‐admission	24.60 ng/mL	0.72 (0.65–0.78)	<0.001	61.70	72.73	0.34
NLRP3 in CSF‐21 days post‐admission	9.69 ng/mL	0.67 (0.60–0.74)	<0.001	52.13	76.14	0.28

Abbreviations: AUC, area under the curve; CI, confidence interval; ROC, receiver operating characteristic.

## 4 DISCUSSION

The crucial role of inflammatory responses in the progression and prognosis of TBI has garnered substantial attention recently and the present study set out to evaluate the inflammatory marker NLRP3, not only as a potential indicator of the severity of TBI but also as a predictor of patient prognosis. Our results clearly indicated that patients with higher levels of NLRP3, particularly in the CSF on the third day post‐injury, are more likely to have an unfavorable prognosis. This observation reinforces the importance of early intervention and close monitoring during the initial days following a traumatic injury. Elevated levels of NLRP3 in the early days after the injury might be indicative of increased inflammation, which can exacerbate neural damage, edema, and other secondary injury processes.

Interestingly, although NLRP3 levels in serum showed variations between day 1 and day 21, those in CSF consistently displayed significant differences between patients with good and poor prognoses. This sustained difference in CSF NLRP3 levels underscores the role of central inflammatory processes in determining TBI outcomes. The CSF, being in direct contact with the brain, might offer a more accurate reflection of cerebral inflammatory processes than peripheral serum samples.

The ROC analysis further bolstered our findings by revealing the predictive potential of NLRP3 levels. Such predictive models can assist clinicians in providing tailored care and interventions to those at higher risk of poor outcomes.

Given the heterogeneous nature of TBI, there has been a growing interest in the identification of objective biomarkers that can predict its severity and prognosis. Our study is preceded by the characterization of a number of serum or CSF biomarkers for TBI prognosis. For example, one of the most extensively studied biomarkers for TBI, S100 Calcium‐binding protein B (S100B),^20^ is primarily found in astrocytes. Increased serum levels of S100B after TBI have been correlated with injury severity and poor outcomes. However, its role as a definitive prognostic marker is still under debate due to its presence in other tissues outside the brain. Another astrocyte protein, Glial fibrillary acidic protein (GFAP) has demonstrated promise as a potential biomarker for TBI. The studies have reported elevated GFAP levels in the blood of TBI patients, with levels correlating with the injury's severity and radiological findings.^21^ Ubiquitin C‐Terminal Hydrolase L1 (UCH‐L1), when detected in blood after brain injury, has been associated with neurological decline and unfavorable outcomes in TBI patients. Its combined assessment with GFAP has been approved by the FDA for TBI diagnosis.^22^ Additionally, increased levels of neurofilament light (NfL) in CSF and serum have been associated with axonal injury and have been studied as potential markers for TBI severity and prognosis.^23^ By demonstrating the predictive value of NLRP3 in serum and CSF, our study provides a comprehensive characterization of NLRP3 as a biomarker, which could be analyzed in clinics more easily than radiological tools, to potentially improve manage of TBI patients by serving as a surrogate or supplemental tool. Notably, NLRP3 may also be used in conjugation with the previously reported biomarkers to further enhance performance of predicting TBI severity.

Our study does have certain limitations. The mechanisms through which NLRP3 affects the progression of TBI were not explored in‐depth. Future studies can delve into the molecular pathways and ascertain the cause‐and‐effect relationship between NLRP3 elevation and TBI prognosis. Also, the inclusion of a larger, more diverse patient cohort could further validate and refine our results.

## 5 CONCLUSIONS

We demonstrated that serum and CSF NLRP3 levels are associated with the severity and prognosis of TBI patients. Predictive and diagnostic tools developed based on NLRP3 could pave the way for improved management of TBI patient and ultimately lead to better outcomes.

## FUNDING INFORMATION

This work was supported by the Project of Ningbo Leading Medical and Health Discipline (2022‐F04); Project of Ningbo Municipal Bureau of Science and Technology (2021S172); National Natural Science Foundation of China (82171366); Key Research and Development Project of Jiangxi Provincial Department of Science and Technology (20203BBGl73172) and the Scientific Research Project of Jiangxi Provincial Department of Education (GJJ200165).

## CONFLICT OF INTEREST STATEMENT

The authors declare that there is no conflict of interest associated with this publication and there has been no significant financial support for this work that could have influenced its outcome.

## Data Availability

The data that support the findings of this study are available from the corresponding author upon reasonable request.
